# Supplementary material for: Control of neural crest multipotency by Wnt signaling and the Lin28/let-7 axis
Source: eLife. 2018 Dec 6;7:e40556. doi: 10.7554/eLife.40556 (PMC6301792; doi:10.7554/eLife.40556)
Supplement: Supplementary file 1. — The corresponding figure number for each experiment is also included in the table. [file elife-40556-supp1.docx]

| Experiment | Embryos analyzed (n) | p-value | Figure |
| --- | --- | --- | --- |
|  |  |  |  |
| Constitutive Lin28a expression |  |  |  |
| *RT-PCR* |  |  |  |
| Pax7 | 6 | 0.0013 | Fig 1e |
| FoxD3 | 6 | 0.036 | Fig 1e |
| SOX5 | 6 | 0.001 | Fig 1e |
| Myc | 6 | 0.027 | Fig 1e |
| ETS1 | 6 | 0.075 | Fig 1e |
| Immunohistochemistry |  |  |  |
| Pax7/FoxD3 | 4 |  | Fig S2 |
|  |  |  |  |
| Lin28a knockdown |  |  |  |
| *RT-PCR* |  |  |  |
| Sox10 | 6 | 0.001 | Fig 1j |
| FoxD3 | 6 | 0.0007 | Fig 1j |
|  |  |  |  |
| let-7 sensor activity |  |  |  |
| HH8 | 5 |  | Fig 3b |
| HH10 | 5 | <0.00001 | Fig 3b |
| HH12 | 5 | 0.0012 | Fig 3b |
|  |  |  |  |
| mature let-7 levels |  |  |  |
| let-7a | 6 | 0.011 | Fig 3f |
| let-7b | 6 | 0.009 | Fig 3f |
| let-7c | 6 | 0.0002 | Fig 3f |
| let-7d | 6 | <0.0001 | Fig 3f |
| let-7g | 6 | 0.045 | Fig 3f |
| let-7i | 6 | 0.14 | Fig 3f |
|  |  |  |  |
| let-7 mimic transfection |  |  |  |
| *RT-PCR* |  |  |  |
| Sox10 | 6 | <0.0001 | Fig 3i |
| FoxD3 | 6 | <0.0001 | Fig 3i |
|  |  |  |  |
| Epistatic experiments |  |  |  |
| *Lin28a MO* |  |  |  |
| FoxD3 | 5 | 0.008 | Fig 3l |
| Sox10 | 5 | <0.0001 | Fig 3l |
| *Lin28a MO + Lin28a O/E* |  |  |  |
| FoxD3 | 5 | 0.4 | Fig 3l |
| Sox10 | 5 | 0.17 | Fig 3l |
|  |  |  |  |
| *Lin28a MO + Lin28a mCCHC* |  |  |  |
| FoxD3 | 5 | <0.0001 | Fig 3l |
| Sox10 | 5 | 0.0003 | Fig 3l |
|  |  |  |  |
| *Lin28a MO let-7 sp* |  |  |  |
| FoxD3 | 5 | 0.9 | Fig 3l |
| Sox10 | 5 | 0.021 | Fig 3l |
|  |  |  |  |
| Let-7 sponge HH12 |  |  |  |
| FoxD3 | 5 | 0.0008 | Fig 3m |
| Pax7 | 5 | 0.051 | Fig 3m |
| Myc | 5 | 0.026 | Fig 3m |
| Ets1 | 6 | 0.0035 | Fig 3m |
| Sox5 | 6 | 0.0045 | Fig 3m |
| Lin28a | 5 | 0.015 | Fig 3m |
|  |  |  |  |
| Lin28a LOF |  |  |  |
| *RT-PCR* |  |  |  |
| ETS1 | 6 | <0.0001 | Fig 4e |
| cMYC | 6 | <0.0001 | Fig 4e |
| SOX5 | 6 | <0.0001 | Fig 4e |
| PAX7 | 6 | <0.0001 | Fig 4e |
| TFAP2B | 5 | 0.001 | Fig 4e |
| SOX8 | 5 | 0.0005 | Fig 4e |
| SOX9 | 5 | 0.0011 | Fig 4e |
|  |  |  |  |
| let-7 GOF |  |  |  |
| *RT-PCR* |  |  |  |
| ETS1 | 6 | 0.0006 | Fig 4e |
| cMYC | 6 | 0.0002 | Fig 4e |
| SOX5 | 6 | <0.0001 | Fig 4e |
| PAX7 | 6 | 0.0006 | Fig 4e |
| TFAP2B | 5 | <0.0001 | Fig 4e |
| SOX9 | 5 | 0.0039 | Fig 4e |
| SOX8 | 5 | <0.0001 | Fig 4e |
|  |  |  |  |
| 3'UTR assay |  |  |  |
| *Flow cytometry* |  |  |  |
| FoxD3 | representative embryo | <0.00001 | Fig 4i |
| Sox10 | representative embryo | 0.47152 | Fig 4i |
| Bar graphs |  |  |  |
| Myc | 5 | <0.0001 | Fig 4j |
| Pax7 | 5 | <0.0001 | Fig 4j |
| FoxD3 | 5 | <0.0001 | Fig 4j |
| Sox10 | 5 | 0.19 | Fig 4j |
| Zic1 | 6 | 0.01 | Fig 4j |
|  |  |  |  |
| CRISPR let-7 targeting |  |  |  |
| Pax7 gRNA1 | 6 | 0.01 | Fig 4k |
| Pax7 gRNA2 | 5 | 0.032 | Fig 4k |
| FoxD3 gRNA3 | 5 | 0.0042 | Fig 4k |
| FoxD3 gRNA4 | 5 | 0.086 | Fig 4k |
|  |  |  |  |
| Wnt1+Wnt4 MO |  |  |  |
| *RT-PCR* |  |  |  |
| Lin28a | 6 | <0.0001 | Fig 5h |
| Lin28E1 | 6 | <0.0001 | Fig 5h |
| let-7a | 5 | 0.0006 | Fig 5h |
| let-7c | 5 | 0.0091 | Fig 5h |
|  |  |  |  |
| Wnt1 overexpression |  |  |  |
| *RT-PCR* |  |  |  |
| Axin2 | 4 | <0.0001 | Fig 5m |
| Lin28a | 5 | 0.024 | Fig 5m |
| Pax7 | 5 | 0.0094 | Fig 5m |
| FoxD3 | 5 | 0.0002 | Fig 5m |
| let-7 sensor | 4 | 0.0034 | Fig 5m |
|  |  |  |  |
| Lin28a O/E |  |  |  |
| FoxD3 | 50 cells | <0.0001 | Fig S2 |
| Pax7 | 50 cells | <0.0001 | Fig S2 |
|  |  |  |  |
| Lin28a O/E |  |  |  |
| *RT-PCR* |  |  |  |
| Barx2 | 5 | <0.0001 | Fig S2 |
| Alx1 | 5 | 0.0002 | Fig S2 |
| Runx2 | 5 | <0.0001 | Fig S2 |
| MASH1 | 6 | 0.0006 | Fig S2 |
| TUJ1 | 7 | 0.0007 | Fig S2 |
| HuC | 7 | <0.0001 | Fig S2 |
| FABP7 | 6 | <0.0001 | Fig S2 |
| GFAP | 6 | <0.0001 | Fig S2 |
|  |  |  |  |
| Lin28a DsiRNA-1 |  |  |  |
| *RT-PCR*    Lin28a | 6 | <0.0001 | Fig S3 |
| FoxD3 | 5 | <0.003 | Fig S3 |
| Sox10 | 6 | <0.0001 | Fig S3 |
| Lin28a DsiRNA-2 |  |  |  |
| *RT-PCR* |  |  |  |
| Lin28a | 6 | 0.0003 | Fig S3 |
| FoxD3 | 6 | 0.0028 | Fig S3 |
| Sox10 | 5 | 0.031 | Fig S3 |
| Lin28 DsiRNA-1  *RT-PCR* |  |  |  |
|  |  |  |  |
| Lin28B | 6 | <0.0001 | Fig S3 |
| FoxD3 | 6 | 0.026 | Fig S3 |
| Sox10 | 6 | 0.03 | Fig S3 |
|  |  |  |  |
| Lin28B DsiRNA-2  *RT-PCR* |  |  |  |
| Lin28B | 6 | <0.0001 | Fig S3 |
| FoxD3 | 6 | 0.27 | Fig S3 |
| Sox10 | 6 | 0.37 | Fig S3 |
|  |  |  |  |
| FoxD3 enhancer vs protein measurements |  |  |  |
| *FoxD3* protein |  |  |  |
| NEAR vs MID |  | <0.0001 | Fig S4 |
| MID vs FAR |  | 0.0006 | Fig S4 |
|  |  |  |  |
| FoxD3-NC2 enhancer |  |  |  |
| NEAR vs MID |  | 0.13 | Fig S4 |
| MID vs FAR |  | 0.55 | Fig S4 |
|  |  |  |  |
| Wnt1 O/E |  |  |  |
| *qRT-PCR* |  |  |  |
| Barx2 | 5 | 0.0017 | Fig S6 |
| Alx1 | 5 | 0.002 | Fig S6 |
| Runx2 | 5 | 0.0001 | Fig S6 |
|  |  |  |  |
|  |  |  |  |
|  |  |  |  |
|  |  |  |  |
