# Supplementary material for: Control of neural crest multipotency by Wnt signaling and the Lin28/let-7 axis
Source: eLife. 2018 Dec 6;7:e40556. doi: 10.7554/eLife.40556 (PMC6301792; doi:10.7554/eLife.40556)
Supplement: Transparent reporting form [file elife-40556-transrepform.pdf]

1st Floor
24 Hills Road
Cambridge CB2 1JP, UK

P 01223 855340
W elifesciences.org
T @elife

eLife's transparent reporting form

We encourage authors to provide detailed information *within their submission* to facilitate the interpretation and replication of experiments. Authors can upload supporting documentation to indicate the use of appropriate reporting guidelines for health-related research (see EQUATOR Network), life science research (see the BioSharing Information Resource), or the ARRIVE guidelines for reporting work involving animal research. Where applicable, authors should refer to any relevant reporting standards documents in this form.

If you have any questions, please consult our Journal Policies and/or contact us: editorial@elifesciences.org.

Sample-size estimation

- You should state whether an appropriate sample size was computed when the study was being designed
- You should state the statistical method of sample size computation and any required assumptions
- If no explicit power analysis was used, you should describe how you decided what sample (replicate) size (number) to use

Please outline where this information can be found within the submission (e.g., sections or figure legends), or explain why this information doesn't apply to your submission:

We defined our sample sizes based on the standards used in the field. The techniques employed in this study have been previously performed in amniote embryos and our sample size were estimated based on published studies. The information regarding sample sizes and statistical analysis for all experiments are detailed in Supplementary Table 1. All functional experiments were performed in single embryos, and at least 5 individual embryos were used in functional assays (each of which serves as a biological replicate). Since the phenotypes observed in each experiment were consistent between our biological replicates, we are confident that we are assaying appropriate number of replicates for each experimental condition.

Replicates

- You should report how often each experiment was performed
- You should include a definition of biological versus technical replication
- The data obtained should be provided and sufficient information should be provided to indicate the number of independent biological and/or technical replicates
- If you encountered any outliers, you should describe how these were handled
- Criteria for exclusion/inclusion of data should be clearly stated
- High-throughput sequence data should be uploaded before submission, with a private link for reviewers provided (these are available from both GEO and ArrayExpress)

Please outline where this information can be found within the submission (e.g., sections or figure legends), or explain why this information doesn't apply to your submission:

eLife Sciences Publications, Ltd is a limited liability non-profit non-stock corporation incorporated in the State of Delaware, USA, with company number 5030732, and is registered in the UK with company number FC030576 and branch number BR015634 at the address 1st Floor, 24 Hills Road, Cambridge CB2 1JP | August 2014

For our experiments, we considered each embryo as a biological replicate. For all qPCR studies the CT value for each gene was measured thrice (for each replicate) which we consider as technical replicates.
For immunohistochemistry, we stained 1- 3 embryos at a time, and hence performed each experiment separately at least thrice , which should account for technical variations. We have detailed the number of replicates for each experiment in supplementary table 1. All the bar graphs shown in the figures include all the biological replicates, and no data was excluded.
If the experiments had outliers, they were represented as box plots or dot plots and the outliers are shown.
Our study contains no high throughput data.

Statistical reporting

- Statistical analysis methods should be described and justified
- Raw data should be presented in figures whenever informative to do so (typically when N per group is less than 10)
- For each experiment, you should identify the statistical tests used, exact values of N, definitions of center, methods of multiple test correction, and dispersion and precision measures (e.g., mean, median, SD, SEM, confidence intervals; and, for the major substantive results, a measure of effect size (e.g., Pearson's r, Cohen's d)
- Report exact p-values wherever possible alongside the summary statistics and 95% confidence intervals. These should be reported for all key questions and not only when the p-value is less than 0.05.

Please outline where this information can be found within the submission (e.g., sections or figure legends), or explain why this information doesn't apply to your submission:

We have reported the statistical tests used in our Methods section. This section also includes the justification for the tests that were employed.
We present the N for each experiment and the corresponding p values in Supplementary Table 1. Additional, in our figure legends we have included what each error bar represents.

(For large datasets, or papers with a very large number of statistical tests, you may upload a single table file with tests, Ns, etc., with reference to sections in the manuscript.)

Group allocation

- Indicate how samples were allocated into experimental groups (in the case of clinical studies, please specify allocation to treatment method); if randomization was used, please also state if restricted randomization was applied
- Indicate if masking was used during group allocation, data collection and/or data analysis

Please outline where this information can be found within the submission (e.g., sections or figure legends), or explain why this information doesn't apply to your submission:

For all our functional experiments, we compared treatment vs control phenotype within the same embryo as has been detailed in our Methods. Thus no group allocation was necessary.

Additional data files ("source data")

eLife Sciences Publications, Ltd is a limited liability non-profit non-stock corporation incorporated in the State of Delaware, USA, with company number 5030732, and is registered in the UK with company number FC030576 and branch number BR015634 at the address 1st Floor, 24 Hills Road, Cambridge CB2 1JP | August 2014

- We encourage you to upload relevant additional data files, such as numerical data that are represented as a graph in a figure, or as a summary table
- Where provided, these should be in the most useful format, and they can be uploaded as "Source data" files linked to a main figure or table
- Include model definition files including the full list of parameters used
- Include code used for data analysis (e.g., R, MatLab)
- Avoid stating that data files are "available upon request"

Please indicate the figures or tables for which source data files have been provided:

Our manuscript did not generate any genomic datasets. We have included source data files for all experiments which were quantified. In our merged source data file, we have included raw data for the following figures:
Figure 1 e, j, l
Figure 3 f, I, l and m
Figure 4 b, c, e, k
Figure 5 h, I, k and l
